# Phenotypic convergence in bacterial adaptive evolution to ethanol stress

**DOI:** 10.1186/s12862-015-0454-6

**Published:** 2015-09-03

**Authors:** Takaaki Horinouchi, Shingo Suzuki, Takashi Hirasawa, Naoaki Ono, Tetsuya Yomo, Hiroshi Shimizu, Chikara Furusawa

**Affiliations:** Quantitative Biology Center (QBiC), RIKEN, 6-2-3 Furuedai, Suita, Osaka 565-0874 Japan; Department of Bioengineering, Tokyo Institute of Technology, 4259 Nagatsuta-cho, Midori-ku, Yokohama 226-8501 Japan; Graduate School of Information Science and Technology, Osaka University, 1-5 Yamada-oka, Suita, Osaka 565-0871 Japan; Graduate School of Information Science, Nara Institute of Science and Technology, Ikoma, Nara 630-0192 Japan; Graduate School of Frontier Biosciences, Osaka University, 1-5 Yamada-oka, Suita, Osaka 565-0871 Japan

## Abstract

**Background:**

Bacterial cells have a remarkable ability to adapt to environmental changes, a phenomenon known as adaptive evolution. During adaptive evolution, phenotype and genotype dynamically changes; however, the relationship between these changes and associated constraints is yet to be fully elucidated.

**Results:**

In this study, we analyzed phenotypic and genotypic changes in *Escherichia coli* cells during adaptive evolution to ethanol stress. Phenotypic changes were quantified by transcriptome and metabolome analyses and were similar among independently evolved ethanol tolerant populations, which indicate the existence of evolutionary constraints in the dynamics of adaptive evolution. Furthermore, the contribution of identified mutations in one of the tolerant strains was evaluated using site-directed mutagenesis. The result demonstrated that the introduction of all identified mutations cannot fully explain the observed tolerance in the tolerant strain.

**Conclusions:**

The results demonstrated that the convergence of adaptive phenotypic changes and diverse genotypic changes, which suggested that the phenotype–genotype mapping is complex. The integration of transcriptome and genome data provides a quantitative understanding of evolutionary constraints.

**Electronic supplementary material:**

The online version of this article (doi:10.1186/s12862-015-0454-6) contains supplementary material, which is available to authorized users.

## Background

Biological systems possess the ability to adapt to environmental changes, which can generate a variety of phenotypes and genotypes. Such emergence of phenotypic and genotypic diversity is considered a result of stochastically fixed genomic mutations during the process of evolution. A question that arises here is whether the process of evolution allows arbitrary phenotypic changes or whether there are constraints that restrict possible variations in phenotypes [1]. The pioneering studies by Waddington [2], which have been corroborated by several other studies, suggests the latter, i.e., constraints on evolutionary dynamics is ubiquitous. One example of such evolutionary constraint is that the earliest embryo of various organisms shows a conserved morphological pattern called the phylotypic period, which is a constrained distribution of phenotype [3]. Here, the relationship between evolutionary constraints and phenotypic plasticity without genetic alteration has generated significant attention [4–7]. However, despite the recognized importance of characterizing evolutionary constraints, quantitative understanding of the process still remains unclear. For this purpose, greater analysis is needed on phenotypic and genotypic changes in a variety of evolutionary courses.

Laboratory evolution of bacteria is a powerful tool to trace phenotypic and genotypic changes in adaptive evolution in a quantitative manner. Recent advances in high-throughput sequencing have made it possible to identify and study fixed mutations in whole-genomic sequences during microbial adaptive evolution. For example, several mutations were identified as beneficial in adaptively evolved *Escherichia coli* (*E. coli*) strains that used glycerol as the carbon source [8]. Other studies using laboratory evolution and genome resequencing have provided evidence that genomic mutations contribute to adaptive phenotypic changes against various environments, including several carbon sources [9–11], different temperatures [12, 13], and the presence of antibiotics [14, 15]. The advancements of genome-wide analysis in laboratory evolution open the door to integrate quantitative data of phenotypic and genotypic changes, which can shed light on the nature of evolutionary dynamics including quantitative understanding of evolutionary constraints.

In this study, we analyzed phenotypic and genotypic changes in the laboratory evolution of *E. coli* cells. In the previous study of laboratory evolution under the ethanol stress condition [16], we found that the overall gene expression changes before and after long-term cultivation were similar among independently evolved tolerant strains. However, it is still unclear relationship between phenotypic change and genetic change during evolution. In this study, first to further analyze the relationship in phenotypic changes in the independently evolved tolerant strain, we quantified time-series of expression changes. The changes of metabolite concentrations were also quantified in the tolerant strains. Then, we assessed genotypic changes in the tolerant strains using high-throughput sequencers, to analyze the relationship between fixed mutations and phenotypic changes. To quantitatively evaluate the effects of fixed mutation on the ethanol tolerance, we introduced all the identified mutations in the genome of the parent strain into one of the tolerant strains. By integrating these phenotypic and genotypic data, we analyzed how the phenotype-genotype mapping is organized in the process of adaptive evolution.

## Results

### Time-series expression analysis in adaptive evolution under ethanol

We previously obtained 6 independently evolved ethanol tolerant *E. coli* strains (A through F) by culturing cells under 5 % ethanol stress for about 1000 generations and found a significantly larger growth rate than the parent strains [16]. Here, we defined "ethanol tolerance" as a state with significantly higher growth rate under 5 % ethanol stress condition, and the term "strain" is used for the mixed population without single-colony isolation. To elucidate the phenotypic changes that occurred during adaptive evolution, we first quantified the time-series of the expression changes by microarray analysis. Starting from frozen stocks obtained at 6 time points in laboratory evolution (0, 384, 744, 1224, 1824, and 2496 h after starting the culture), cells were cultured under 5 % ethanol stress, and mRNA samples were obtained in the exponential growth phase for microarray analysis (quantified expression data are presented in Additional file 1: Table S1).

**Fig. 1:**
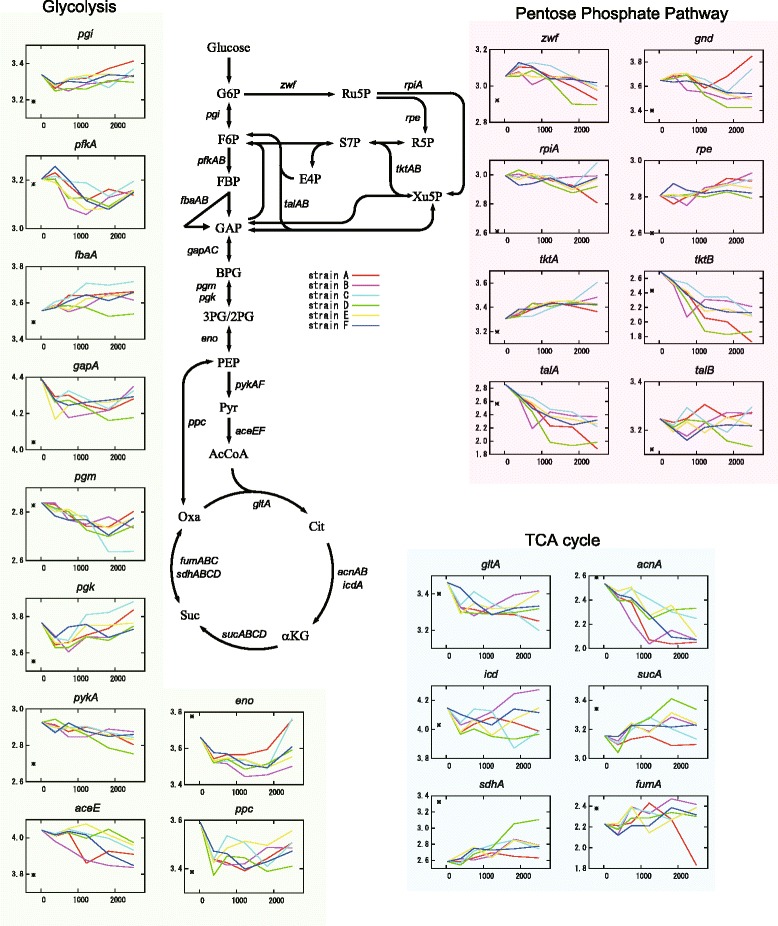
Time-series transcriptome analysis for adaptive evolution of *E. coli* to ethanol stress. Expression changes of representative genes in the central metabolic pathway including glycolysis, the pentose phosphate pathway, and TCA cycle are shown. In each inset, the horizontal axis shows time (hours), and the vertical axis shows expression level (a.u.). Expression levels of 0 h in each gene represent the ones of parent strain. Asterisks (*) indicate expression levels of parent strain obtained without adding ethanol as a reference. The numbers of generations from 0 to 2000 h were strain A:1025, B:1002, C:950, D:990, E:954, and F:938, respectively. Abbreviations: 2PG, 2-Phosphoglyceric acid; 3PG, 3-phosphoglycerate; AcCoA, acetyl-CoA; αKG, α-ketoglutarate; BPG, 1,3-bisphosphoglycerate; Cit, citrate; E4P, erythrose4-phosphate; F6P, fructose 6-phosphate; FBP, fructose 1,6-bisphosphate; GAP, glyceraldehyde 3-phosphate; G6P, glucose 6-phosphate; Oxa, oxaloacetate; PEP, phosphoenolpyruvate; Pyr, pyruvate; R5P, ribose 5-phosphate; S7P, sedoheptulose 7-phosphate; Suc, succinate; X5P, xylulose 5-phosphate

The results of the time-series transcriptome analysis revealed that the expression changes during adaptive evolution were similar among tolerant strains. For example, Fig. 1 shows the expression changes of genes in the central metabolic pathway including glycolysis, the tricarboxylic acid (TCA) cycle, and the pentose phosphate pathway. Interestingly, common expression changes were not always monotonic (e.g., *pfkA* gene) over time, but were rather synchronized complex expression changes on a much longer time-scale than the generation time observed. Additionally, a common and gradual up-regulation of genes involved in some amino acid biosynthesis pathways was also observed (Additional file 2: Figure S1). Our previous work suggests that these pathways might contribute to ethanol tolerance [16].

**Fig. 2:**
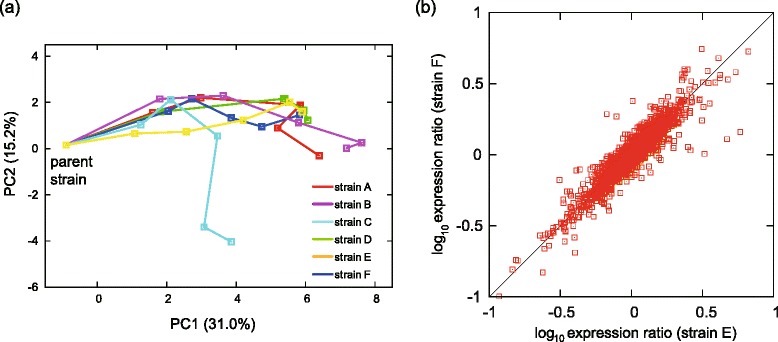
Similarity among expression changes in the tolerant strains. **a** Changes in PCA scores during adaptive evolution. Starting from the parent strain, changes in the expression profiles during adaptive evolution are plotted as orbits in the two-dimensional PCA plane. **b** An example of the correlation between expression changes that occurred in two tolerant strains. The expression changes in strain E and F were plotted. Horizontal and vertical axes are log-transformed expression ratios with the parent strain, and each dot represents the expression change of the gene. All possible combinations are shown in Additional file 3: Figure S2

Figure 2a shows overall expression changes during the adaptive evolution of the six tolerant strains by principal component analysis (PCA). The orbits in PCA space, which represent expression profile changes, were similar except for strain C. The reason for this exception might be that strain C has an approximately 200 kbp region in the genome that was duplicated (discussed below), and the expression levels of genes in this region were increased by this duplication. The expression analysis also demonstrated that the overall expression changes between the parent and tolerant strains at the endpoint were similar (Fig. 2b and Additional file 3: Figure S2). These results indicated that even though these strains adapted to ethanol stress in independent cultures, the expression profiles converged into almost identical adapted states with similar orbits of expression changes.

### Metabolome analysis of ethanol tolerant strains

**Fig. 3:**
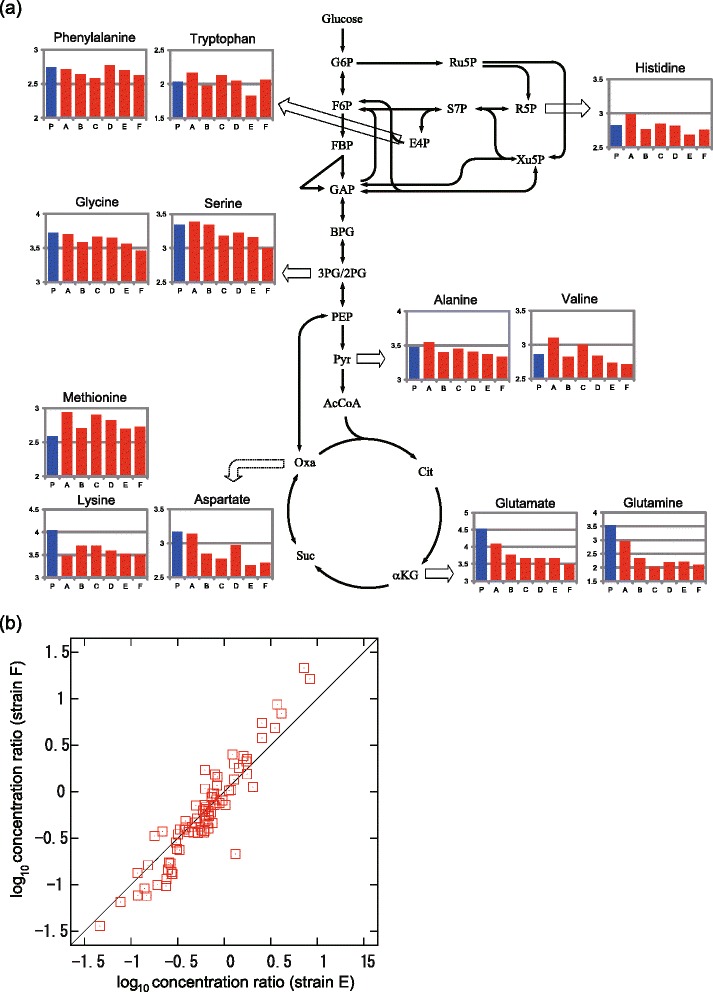
Metabolome analysis of ethanol tolerant *E. coli* strains under ethanol stress conditions. **a** Concentration of amino acids in ethanol tolerant strains. In each inset, the vertical axis shows the log-transformed absolute concentration (μM). The blue bar and red bars indicate the data of the parent strain and tolerant strains, respectively. **b** Correlation between metabolite concentration changes in strains E and F. Horizontal and vertical axes represent log-transformed concentration ratios with the parent strain, and each dot represents the concentration change of the metabolites

To further characterize the phenotypic changes that occurred in the tolerant strains, we measured metabolite concentration changes between parent and tolerant strains. Using capillary electrophoresis time-of-flight mass spectrometry (CE-TOFMS), we quantified the intracellular concentrations of 83 metabolites (complete data are presented in Additional file 4: Table S2). The intracellular concentrations of some amino acids in the parent and tolerant strains are presented in Fig. 3a. These concentrations generally decreased in the tolerant strains, except for that of methionine. The decrease was especially true for amino acids that originated from precursors in the tricarboxylic acid (TCA) cycle. This suggests a change of metabolic state in the TCA cycle in tolerant strains, a conclusion supported by the significant decrease in the expression of genes related to the TCA cycle (Fig. 1). For example, glutamate acts as a major amino-group donor in amino acid biosynthesis, and thus a decrease in its concentration can cause a decrease in the concentration of other amino acids. The decrease in amino acid concentration can activate the amino acid starvation response, which is consistent with the up-regulation of genes related to amino acid biosynthesis. In contrast, the concentrations of metabolites in purine metabolism generally increased (Additional file 5: Figure S3). This concentration increase might be caused by the up-regulation of genes involved upstream of the purine biosynthesis pathway (Additional file 6: Figure S4), by which phosphoribosyl pyrophosphate (PRPP), the precursor for purine nucleotide synthesis produced from ribose-5-phosphate, is converted into inosine 5'-monophosphate (IMP). No significant concentration change was observed for metabolites in pyrimidine metabolism.

The metabolome analysis also demonstrated similar changes in metabolite concentration among the tolerant strains, which resembles observations for gene expression changes. The correlation of overall metabolite concentration changes between independently obtained tolerant strains indicated similar metabolite shifts (Fig. 3b and Additional file 7: Figure S5). Both the transcriptome and metabolome analyses showed that phenotypic changes were similar among tolerant strains even though they were obtained from independent long-term cultivations.

### Genome resequencing analysis of ethanol tolerant strains

Genotype changes in each tolerant strain were analyzed using two high-throughput sequencers, SOLiD and Illumina MiSeq (see Methods for details). In the resequencing analysis, we extracted genomic DNA samples from the cell population at the end-point of the experimental evolution without single-colony isolation, to identify genotype changes that were fixed in the majority of tolerant cells and to avoid a fixation of minority mutations. For point mutations, SOLiD and Illumina analyses identified 136 and 138 fixed mutations in all 6 tolerant strains, respectively, with 135 of these mutations being identified in both analyses. The discrepancy (4 point mutations in strain A) was checked by Sanger sequencing, and it was confirmed that 3 were true positives and the other a false positive. After screening indels by SOLiD sequencing, we identified 7 small (< 500 bp) and 13 large indels in all tolerant strains. We verified these small and large indels by Sanger sequencing, finding all were true positives. Finally, in strain C, the coverage of an approximately 200 kbp region was significantly higher than in other strains (Additional file 8: Figure S6), which strongly suggested that the corresponding region duplicated during long-term cultivation.

The identified mutations at the end-point of experimental evolution are summarized in Table 1. The number of mutations in strain A was significantly higher than other strains (Additional file 9: Table S3). This was likely due to a mutation leading to a stop codon in the *mutS* coding region, which codes a mismatch repair protein. It is known that disruption of *mutS* significantly increases the mutation rate of *E. coli* cells [17]. We confirmed that there were only 3 mutations in strain A at 1512 h (about 600 generations) after commencing laboratory evolution and these did not include a mutation in *mutS*. This result suggested that after 1512 h, the mutation in the *mutS* gene was fixed and resulted in a significant increase in the mutation rate. The emergence of a strain with a significantly high mutation rate, or a “mutator,” is often observed in the laboratory evolution of microorganisms [18–21].

**Table 1:** List of identified mutations

Strain	Type	Gene	Position		Nucleotide change		Source	Gene Description
			Reference	Gene				
A	125 SNPs and 6 Indels (see Additional file 9: Table S3)							
B	Ins	***hns*** ^**a**^	1294843	−273	1195 bp	IS5 insertion, promoter	SOLiD and Sanger	global DNA-binding transcriptional dual regulator
	Ins	*yeaR*	1881551	147	1342 bp	IS186 insertion	SOLiD and Sanger	conserved hypothetical protein
	SNP	***iscR***	2660496	292	A → T		SOLiD and MiSeq	DNA-binding transcriptional activator
	SNP	*ilvG*	3685148	974	A → T		SOLiD and MiSeq	acetolactate synthase II, large subunit
C	Del	12 genes	575013		−6775 bp		SOLiD and Sanger	*insH,nmpC,essD,ybcS,rzpD,rzoD,borD,ybcV,ybcW,nohB,tfaD,ybcY*
	Ins	*nagE*	705229	864	+3:CCG	3 bp insertion	SOLiD and Sanger	fused N-acetyl glucosamine specific PTS enzyme
	Ins	***hns***	1294843	−273	1195 bp	IS5 insertion, promoter	SOLiD and Sanger	global DNA-binding transcriptional dual regulator
	SNP	*yeaY*	1892079	168	T → A	synonymous	SOLiD and MiSeq	predicted lipoprotein
	Ins	*menC*	2380504	485	1343 bp	IS186 insertion	SOLiD and Sanger	o-succinylbenzoyl-CoA synthase
	SNP	***relA***	2910761	1547	A → G		SOLiD, MiSeq and Sanger	(p)ppGpp synthetase I/GTP pyrophosphokinase
	SNP	*rpoC*	3448513	2819	G → A		SOLiD and MiSeq	RNA polymerase, beta prime subunit
	SNP	*rpoA*	4200347	961	T → A		SOLiD and MiSeq	RNA polymerase, alpha subunit
D	Ins	***hns***	1294843	−273	1195 bp	IS5 insertion, promoter	SOLiD and Sanger	global DNA-binding transcriptional dual regulator
	SNP	*proQ*	1916977	272	A → T		SOLiD and MiSeq	predicted structural transport element
	SNP	*ispG*	2639469	992	T → C		SOLiD and MiSeq	1-hydroxy-2-methyl-2-(E)-butenyl 4-diphosphate synthase
	SNP	*rpsD*	4198966	226	T → G		SOLiD and MiSeq	30S ribosomal subunit protein S4
	Ins	*yjhA*	4544220	−39	1199 bp	IS5 insertion, promoter	SOLiD and Sanger	N-acetylnuraminic acid outer membrane channel protein
E	Ins	***hns***	1294843	−273	1195 bp	IS5 insertion, promoter	SOLiD and Sanger	global DNA-binding transcriptional dual regulator
	Ins	***cspC***	1909109	45	1199 bp	IS5 insertion	SOLiD and Sanger	stress protein, member of the *CspA* (cold shock protein) family
	SNP	***relA***	2910944	1364	A → T		SOLiD, MiSeq and Sanger	(p)ppGpp synthetase I/GTP pyrophosphokinase
	Ins	*yhcM*	3379114	739	+1:C		SOLiD, MiSeq and Sanger	hypothetical protein with nucleoside triphosphate hydrolase domain
	SNP	*atpE*	3715545	54	T → G		SOLiD and MiSeq	F0 sector of membrane-bound ATP synthase, subunit c
F	Del	*miaB*	694563	728	−88 bp		SOLiD, MiSeq and Sanger	isopentenyl-adenosine A37 tRNA methylthiolase
	Ins	***cspC***	1909109	45	1199 bp	IS5 insertion	SOLiD and Sanger	stress protein, member of the *CspA* (cold shock protein) family
	SNP	*wzxC*	2120992	1304	A → T		SOLiD, MiSeq and Sanger	colanic acid exporter
	SNP	***iscR***	2660468	320	T → A		SOLiD, MiSeq and Sanger	DNA-binding transcriptional activator
	SNP	***relA***	2911891	417	T → G		SOLiD, MiSeq and Sanger	(p)ppGpp synthetase I/GTP pyrophosphokinase

^a^Bold-faced genes represent overlap among evolved strains

In contrast to the more than one hundred fixed mutations in strain A, the number of fixed mutations was relatively lower in the other strains. As mentioned above, the phenotypic changes that occurred in independently evolved tolerant strains were similar, which might suggest mutations fixed in identical or related genes contributed to the changes. We found that mutations were commonly fixed in *relA*, which codes guanosine tetraphosphate synthetase. RelA regulates a stringent response by producing guanosine tetraphosphate (ppGpp) [22]. The stringent response is widely observed in bacteria as a stress response in reaction to nutrient starvation [23] or various environmental stresses [24]. When *E. coli* face such stresses, growth-related activities including replication, transcription, and translation are tightly inhibited, which are triggered by the accumulation of ppGpp. Thus, the mutations commonly fixed in *relA* may relax the stringent response caused by ethanol stress to recover growth activity. The mutations in *relA* and *spoT*, which codes an enzyme that plays a major role in ppGpp degradation, have been widely observed in the laboratory evolution of *E. coli* under various conditions, including glucose limitation [25] and high temperature [13]. Here, relaxing the stringent response by mutating the *relA* and *spoT* genes may increase fitness under stress. Furthermore, in strains A, B, C, D, and E, insertion sequence element 5 (IS5) was inserted into the promoter region of *hns*, which codes for a DNA binding protein that has various effects on gene expression [26]. However, no significant change in *hns* expression was observed in these strains. Except for *relA* and *hns*, no functional overlap among the mutations fixed in more than two tolerant strains was determined.

### Fitness contribution of fixed mutations

**Fig. 4:**
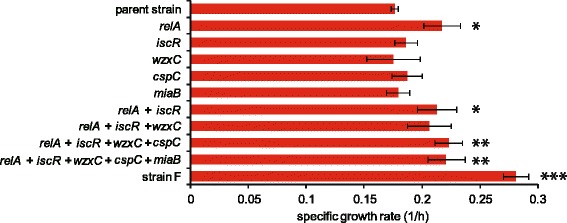
Growth rates of site-directed mutants with ethanol stress. All mutations identified in strain F were introduced back to the parent strain. For each mutant, the names of the mutated genes are shown. Among the mutations identified in strain F, the mutations for *cspC* and *miaB* correspond to IS insertion and 88 bp deletion, respectively, while other mutations were SNPs. Error bars indicate standard deviations calculated from three independent cultures. Different asterisks (*) indicate significant differences (*, *P* < 0.05; **, *P* < 0.01; ***, *P* < 0.001), which were determined by Dunnett’s test compared with parent strain

To evaluate the contribution of fixed mutations to the growth increase under ethanol stress, we introduced all identified mutations in strain F into the parent genome by site-directed mutagenesis [27]. We selected strain F for this analysis because the number of IS insertion was smallest among the tolerant strains. In strain F, we identified 5 mutations, including 3 single nucleotide substitutions, one small deletion, and one insertion in the genome of strain F. The sequence of the 1199-bp insertion was identical to IS5, which disrupted the ORF of *cspC* . Since the insertion of IS5 into the same position of the parent genome was difficult experimentally, we constructed a *cspC* deletion strain. Figure 4 shows the growth rates of the constructed strains by site-directed mutagenesis measured under the ethanol stress condition. Here, the main purpose of the analysis was to introduce all identified mutations in strain F into the genome of the parent strain, instead of constructing mutant strains with all possible combinations of mutations. Thus, the combinations of 2, 3, and 4 mutations are arbitrary chosen to optimize the speed to construct the strain with 5 mutations. The results in Fig. 4 demonstrated that the fixed mutation in *relA* significantly contributed to the growth rate increase (*P* < 0.05; determined by Dunnett’s test between parent and the reconstructed strains after one-way ANOVA). However, other mutations had no significant effect on the growth rate, and even when all fixed mutations in strain F were introduced into the parent genome, the observed growth rate of the mutated strain was significantly smaller than that of strain F under the ethanol stress condition.

### Timing of fixed mutations

**Fig. 5:**
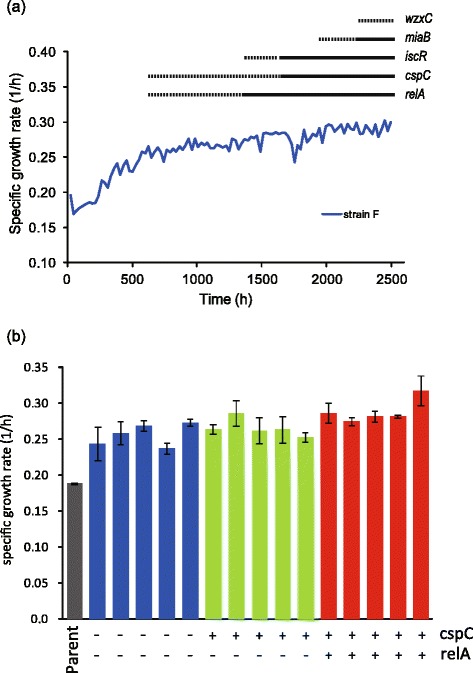
Time-series analysis of the mutation fixation. **a** Timing of mutation fixation events in strain F. To identify the timing of mutation fixation, genomic DNA samples obtained at 12 different time points (216, 384, 576, 744, 888, 1056, 1224, 1392, 1584, 1824, 1968, and 2232 h after inoculation) were applied to Sanger sequencing. For each of the 5 identified mutations, the results of the Sanger sequencing is presented as a solid or dotted line. The solid line indicates that the mutation was fixed in the population at the corresponding time point, while the dashed line indicates the case of two peak signals, which indicates polymorphism in the population with and without the mutation. For example, in cells at 576 h after inoculation, only some have mutations in *relA* and *cspC.* **b** Specific growth rates of cloned *E. coli* cells with and without *cspC* and *relA* mutations under ethanol stress condition. Each bar represents the specific growth rate of an isolated clone, which were obtained from strain F cell populations at 576 h. " + " and "-" mean with and without the corresponding mutation, respectively. Blue, green, and red bars represent the growth rates of clones without mutations, that with *cspC* mutation only, and with *cspC* and *relA* mutations, respectively. Error bars indicate standard deviations calculated from three independent cultures

To further evaluate the contribution of the fixed mutations on ethanol tolerance, we analyzed the relationship between the growth increase under ethanol stress and the timing of mutation-fixation events in long-term cultivation in strain F. To identify the timing, genomic DNA samples obtained from cell populations that had heterogeneous genotypes at 12 different time points were applied to Sanger sequencing. Therefore, in some cases, the peak signals in the Sanger sequencing revealed mixed populations, i.e., cells with and without a specific mutation coexisted in the population. Figure 5a shows the time the mutations in strain F emerged. The increased growth rate did not always correlate with fixation events. More importantly, although at 576 h after inoculation no mutation was fixed in the majority of the cell population, the growth rate under ethanol stress significantly increased. Some cells at 576 h had mutations in the *relA* and *cspC* genes that may have contributed to the observed growth increase. To confirm this possibility, we isolated 48 clones from the cell population at 576 h and analyzed fixed mutations in *relA* and *cspC* by Sanger sequencing. Among the 48 clones, (i) 5 had both *relA* and *cspC* mutations, (ii) 6 had the *cspC* mutation only, and (iii) the other 37 clones had no mutation. To evaluate the effect of these mutations on the population at 576 h, we randomly selected 5 clones from the groups (i), (ii), and (iii), and measured the growth rates of clones with and without mutations under the ethanol stress condition. As shown in Fig. 5b, clones with or without *relA* and *cspC* mutations showed significantly larger growth rates than parent strain (*P* < 0.001; determined by Dunnett’s test between parent and other clones after one-way ANOVA), and there was no significant growth difference between clones. It should be noted that, the average growth rates of clones corresponding to groups (i), (ii), and (iii) were significantly higher than the clone in which 5 identified mutations were introduced (Fig. 4). These results suggested that, the effect of these mutations into the parent genome was smaller than the increase of growth rate in the cultivation from 216 to 576 h, even though in some clones no mutations was suggested to be fixed on the genome.

The results in Fig. 5a and 5b suggested the possibility of clonal interference. The result in Fig. 5b suggested that *cspC* mutation was fixed on a cell with *relA* mutation, while the result in Fig. 5a suggested *relA* mutation rose to prominence before *cspC* mutation. These results might suggest that, in the evolutionary dynamics of these populations, there were multiple clones with different beneficial mutations coexisting and competing in the environment, i.e., clonal interference [28].

### Stability of ethanol tolerance

We reasoned that if the observed ethanol tolerance is due to phenotypic plasticity without genetic alteration, then the phenotype of the ethanol tolerance would likely be unstable when the environment changes. We therefore cultivated cells with ethanol tolerance in an ethanol-stress free environment for 200 generations. Two cell populations were used: strain F and the cell population obtained at 576 h in the cultivation of strain F. After cultivation in the non-stress condition, we measured the growth rate under 5 % ethanol stress to evaluate the stability of the ethanol tolerance. Ethanol tolerance was stably maintained even after 200 generations (Additional file 10: Figure S7), which suggests that the observed phenotypic changes in the tolerant strains were stably memorized and passed on to progeny cells.

### Growth evaluation of clones in the population of the ethanol tolerant strains

In this study, to analyze the characteristics of the majority of cell population, we measured mixed population without single colony isolation when we performed transcriptome, metabolome, and genome resequencing analysis. To evaluate the effect of clonal interference in the population of the ethanol tolerant strains, we isolated clones from the end-point population of strains E and F. Then, we quantified the growth rate of these clones under the ethanol stress condition as shown in Additional file 11: Figure S8. The result demonstrated that there were no difference between isolated clones and mixed population, suggesting that the effect of clonal interference is negligible in the population of ethanol tolerant strain we obtained.

## Discussion

Transcriptome and metabolome analyses revealed that phenotypic changes that occurred in ethanol tolerant strains were similar among independently evolved strains. Gene expression changes over time were found to exhibit high similarity among tolerant strains, which included non-monotonic expression changes with time scales much longer than the generation time. The observed phenotypic convergence to similar orbits clearly demonstrates the existence of evolutionary constraints in the adaptive evolution dynamics.

Using high-throughput sequencers, we identified fixed mutations in the tolerant strains. One tolerant strain had a significantly higher number of fixed mutations than the others, probably due to disruption of *mutS*, which is involved in the mismatch repair mechanism. For the other tolerant strains, the number of fixed mutations was less than 10. We found that these mutations were commonly observed in the *relA* gene, which is involved in stringent response via ppGpp production, suggesting that the stringent response triggered by the ethanol stress was relaxed by these mutations in the tolerant strains and therefore did not diminish growth activity as would otherwise be expected. These mutations could be regarded as candidate beneficial mutations for ethanol tolerance.

The observed synchronized slow expression changes might suggest the existence of deterministic slow dynamics of adaptive evolution, and can at the same time be difficult to explain by phenotypic changes caused by a small number of mutations. In fact, by analyzing the timing of the mutation fixation, we demonstrated that the increase in growth rate of strain F did not correlate with mutation fixation events, and *E. coli* clones without any identified beneficial mutation grew significantly faster than the parent strain under ethanol stress (Fig. 5). Furthermore, to evaluate the contribution of identified mutations to phenotype, we introduced these mutations into the genome of the parent strain of strain F and then quantified the change in ethanol tolerance. The results showed that the observed ethanol tolerance in strain F could not be reproduced by introducing the identified mutations (Fig. 4). Importantly, the ethanol tolerance was maintained after cultivation of 200 generations under conditions without ethanol stress, which indicated that the phenotype of ethanol tolerance in these strains was somehow stably maintained, although the contribution of fixed mutations to the tolerance was obscure.

One possible explanation of these results is that there are unidentified contributions of genetic alteration to the observed phenotypic changes. In our genome resequencing analysis, the genomic regions with low read coverage (less than 10 reads) were excluded from the mutation identification procedure. Such low coverage regions cover 22,162 base pairs (0.47 % of the genome) in total. Most of the low coverage regions have identical or nearly identical sequences to the other regions of the genome. In fact, 20,831 out of 22,162 (94 %) base pairs in the low coverage regions correspond to the coding region of rRNA, tRNA, and ribosomal proteins, which have several nearly identical sequences on the *E. coli* genome. For such regions, the identification of mutations is difficult by using the short-read sequencing technology. Thus, we cannot exclude that the possibility that there were some mutations in such regions, which were failed to be identified but contributed to the observed ethanol tolerance.

Another possibility is that the growth increase observed in the adaptive evolution experiment under ethanol stress was due to phenotypic plasticity without genetic alteration, and that this plasticity could be stably memorized in the intracellular state and be inherited by progeny cells, a phenomenon called trans-generational plasticity [29, 30]. Several studies demonstrated the existence of trans-generational plasticity in eukaryotic organisms [31–33]. It has recently been shown in bacteria, that trans-generational transmission of cellular components allows responses to environmental change with a memory [34]. However, in such cases, an adapted state is generally maintained only for several generations. In contrast, that ethanol tolerance was maintained in tens of generations in the present study suggests machinery for stable information inheritance. Similar epigenetic memory was also suggested to play a role in the evolution of antibiotic resistance in *E. coli* [35]. In *E. coli* cells, genome methylation patterns are known to act as epigenetic memory that controls the expression profile [36, 37], as well as the binding of histone-like proteins, such as H-NS and Fis, to genomic DNA [38, 39]. These epigenetic mechanisms might contribute to the observed non-genetic memory and should be considered in future studies.

## Conclusion

We analyzed phenotypic and genotypic changes of *E. coli* cells that occurred during adaptive evolution to ethanol stress and found that the evolutionary orbits of phenotype among independent culture series were similar, indicating the existence of evolutionary constraints. The relationship between genetic mutations and phenotypic changes were complex, which might suggest that part of the phenotypic changes were due to contribution of phenotypic plasticity without genetic alterations. The detailed analysis of phenotypic and genotypic changes in this study provides a better understanding of the nature of adaptive evolution, including non-genetic contributions to adaptive phenotypic changes.

## Methods

### Laboratory evolution

The *E coli* strain W3110 was obtained from National BioResource Project (National Institute of Genetics, Japan) and used for all laboratory evolution cultures. Ethanol tolerant strains, A through F, were obtained as previously described [16]. Briefly, cells were grown in 10 ml of M9 minimal medium with 5 % (v/v) ethanol at final concentration. Cell cultures were performed at 30 °C with shaking at 150 strokes min^−1^ using water bath shakers. We diluted the cells in fresh medium every 24 h and maintained an exponential growth phase by adjusting the initial cell concentration. In the daily serial transfer, the population size of the transfer depend on the growth rate, which is within the range of approximately from 3.0 × 10^5^ cells to 3.0 × 10^6^ per test tube. The adaptation of the *E. coli* was evaluated by measuring the optical density of the culture at 600 nm (OD_600_) and calculating specific growth rate using OD_600_ data. The specific growth rate is defined as the increase of cell concentration (OD_600_) divided by cell concentration, by calculating the slope of the logarithmic plot of OD_600_ value [40].

### Transcriptome analysis by microarray

For transcriptome analysis, a custom-designed tilling microarray of *E. coli* W3110 in Affymetrix platform was used. The platform contained approximately 1.5 million perfect-match 21-bp probes for the *E. coli* genome and an approximately 4.5 million of corresponding single-base mismatch probes [41]. All samples including the parent strain and end-point sample was inoculated from the frozen stock into 10 mL of M9 medium for preculture. All samples including the parent strain and end-point sample was inoculated from the frozen stock into 10 mL of M9 medium for preculture. In this study, we did not use published data of the parent and end-point samples in [16], instead we re-analyzed these sample with the other time-series samples to avoid unexpected biases. Five-microliter aliquots of preculture medium cells were inoculated into 10 mL of M9 medium with 5 % (v/v) ethanol and cultured for 10 generations. Cells in the exponential growth phase were harvested by centrifugation and stored at −80 °C before RNA extraction. The time-points at which mRNA samples were collected for transcriptome analysis were 384, 744, 1224, 1824, and 2496 h after starting the culture in each strain, and parent strain was used as data for 0 h. Total 31 samples with adding ethanol condition were prepared. Total RNA was isolated and purified from cells using an RNeasy mini kit with on-column DNA digestion (Qiagen, Hilden, Germany). The synthesis of cDNA, fragmentation and end-terminus biotin labeling were carried out in accordance with Affymetrix protocols. Hybridization, washing, staining, and scanning were carried out according to the Expression Analysis Technical Manual (provided by Affymetrix). To obtain the absolute expression levels of genes from microarray raw data, we used the Finite Hybridization model [42, 43]. Expression levels were normalized using the quantile normalization method [44]. In this study, we used 2420 gene expression levels which were higher than the quantification limit in all samples, and expression data under the quantification limit were discarded. The quantification limit was set to 100 based on our previous study [42]. The reproducibility of the analysis was checked by repetitive experiments using the sample of the parent strain (Additional file 12: Figure S9a), in which all data of repetitive experiments were within the range of 1.7 fold. Information on gene regulation was obtained from RegulonDB [45].

### Metabolome analysis by capillary electrophoresis time-of-flight mass spectrometry

Metabolomic analysis was performed using capillary electrophoresis time-of-flight mass spectrometry (CE-TOFMS). The sample preparation method for CE-TOFMS analysis was previously reported [46]. Briefly, cells in the exponential growth phase were harvested by filtration (Isopore™ Membrane Filters HTTP, Millipore, Billerica, MA) and washed with water. The filter was immersed in methanol containing internal standards to quench metabolic reactions and extract intracellular metabolites before sonication for 30 s. To remove phospholipids, the methanol solution was mixed with chloroform and water and then centrifuged at 4,600 g for 5 min at 4 °C. The separated methanol/water layer was filtered through a 5 kDa cutoff filter (Millipore) by centrifugation at 9,100 g and 4 °C to remove proteins. The filtrate was lyophilized and dissolved in 25 μL of water prior to the CE-TOFMS analysis.

CE-TOFMS analysis was performed using the Agilent 7100 CE system equipped with the Agilent 6224 TOF-MS system, the Agilent 1200 isocratic HPLC pump, the G1603A Agilent CE/MS adapter kit, and the G1607A Agilent CE/MS sprayer kit (Agilent Technologies). For system control and data acquisition, Chemstation software for CE- TOFMS (Agilent Technologies) and MassHunter software (Agilent Technologies) were used. The concentration of each metabolite in methanol was quantified using the relative peak area of each metabolite to the internal standard peak area obtained from biological samples and the relative peak area obtained from chemical standards mixtures that included amino acids; intermediate metabolites from glycolysis, TCA cycle, and PPP (50 μM each); and internal standards including 25 μM methionine sulfone and 25 μM camphor-10-sulfonic acid (Human Metabolome Technologies) analyzed in parallel with experimental samples. Peak area data were obtained using the MassHunter software for qualitative analysis (Agilent Technologies). The reproducibility of the analysis was checked by repetitive experiments using the sample of the parent strain (Additional file 12: Figure S9b), in which all data of repetitive experiments were within the range of 3 fold.

### Genome resequencing

Frozen stocks of the strains were grown overnight in 10 ml of M9 minimal medium at 30 °C. Precultured cells were diluted to OD_600_ 0.05 and grown in 10 mL of fresh M9 medium. When OD_600_ reached approximately 2.0, Rifampicin (final concentration 300 μg/mL) was added to block the initiation of DNA replication, and the culture was continued for another 3 h. The cells were collected by centrifugation at 16,000 × g for 2 min and 25 °C and then the pelleted cells were stored at −80 °C prior to genomic DNA purification. Genomic DNA was isolated and purified using a Wizard® Genomic DNA Purification kit (Promega) in accordance with the manufacturer's instructions. To improve the purity of genomic DNA, additional phenol extractions were performed before and after the RNase treatment step. The purified genomic DNAs were stored at −30 °C prior to use.

The same genomic DNA samples of the parent and ethanol tolerant strains were sequenced using both SOLiD DNA analyzer (Life Technologies) and Illumina MiSeq Desktop Sequencer (Illumina). For SOLiD sequencing, mate-paired libraries (2 × 50 bp) of 1200 bp insert size were generated and sequenced according to the manufacturer's protocol, which resulted in about 200-fold coverage on average. For Illumina sequencing, paired-end libraries (2 × 250 bp) were generated using Nextera v2 technology and sequenced by the MiSeq system according to the manufacturer's protocol (Illumina), which resulted in about 180-fold coverage on average.

For identification of point mutations by SOLiD sequencing, the sequence reads were mapped to the reference genome of *E. coli* W3110 with SOLiD bioscope software (version 1.2.1) (Life Technologies). Point mutations were subsequently called by the diBayes algorithm (Life Technologies), in which the threshold *p*-value was set to 10^−7^. To obtain only those mutations present in the majority of cells, variant calls with a ratio of variant reads less than 0.6 were excluded from further analysis. For Illumina sequence data, the sequence reads were mapped to the reference genome by SSAHA2 [47]. For each potential point mutation, we extracted those with coverage reads more than 10 and a ratio of variant read to wild-type read more than 0.6. When the point mutation calls by these two methods produced discrepancies, the candidate mutations were confirmed by Sanger sequencing.

The identification of small indels (< 500 bp) were performed by SOLiD bioscope software, in which the default parameter setting was used. The small indels identified by SOLiD sequencing and bioscope software were confirmed visually using the mapping of reads obtained by Illumina MiSeq.

For the identification of large indels, we implemented a detection algorithm based on distances between mapped SOLiD mate-paired sequence reads as follows. After removing low quality reads (mapping quality < 10 or including bases with base quality < 30), we mapped mate-paired sequence reads by bioscope software, and then used all mapped read pairs to calculate the mean and standard deviation of the distance between any two mapped reads. When indels are fixed in the genome, the distance between two mapped reads mapped to one region shows a deviation from the other genome region. We screened genomic regions at which the median of the mapped read distances was more than 3 SD from the mean, and the presence of an indel was confirmed visually using the mapping. When the pattern of the read distances suggested an insertion and part of the counterpart reads was mapped to an IS element, an IS element insertion was assumed and validated manually. All indels identified by SOLiD sequencing were also identified by Sanger sequencing, as predicted.

The sequence reads from the parent strains were also mapped to the reference genome of *E. coli* W3110, and mutations were screened by the above methods. Point mutations and indels found in the parent strains (shown in Additional file 13: Table S4) were also found in all tolerant strains and discarded from further analysis.

### Effect of genomic mutations on ethanol tolerance

Each identified mutation was introduced into the parent strain using the suicide plasmid method [27]. This approach enables the introduction of any desired mutation without leaving an antibiotic marker in the genome. DNA fragments including identified mutations were cloned into suicide plasmid pST76-K and inserted into the chromosome of the parent strain. Allele replacement and marker removal was performed using helper plasmid pUC19RP12 (These plasmids were kind gifts from Dr. Gyorgy Posfai, Biological Research Centre of the Hungarian Academy of Sciences, Hungary). To eliminate the helper plasmid, obtained mutants were cultured in M9 medium at 30 °C. Primer information of the mutant construction is summarized in Additional file 14: Table S5. To evaluate the effect of the mutations on ethanol tolerance, the specific growth rate of mutated strains was quantified in M9 medium with 5 % ethanol. The conditions for these cultures were identical to those in laboratory evolution. The cultures of each strain were performed three times independently.

## Availability of supporting data

Both the normalized expression data sets and the raw CEL files were deposited in the NCBI Gene Expression Omnibus database under the GEO Series accession number GSE59050. The mate-pair sequencing data by SOLiD and the paired-end sequencing data by Illumina MiSeq are available from the DDBJ Sequence Read Archive of the DNA Data Bank of Japan (DRA) under accession number DRA002309.

## Additional files

Additional file 1: Table S1. (CSV 876 kb)Time series expression analysis. The expression levels of 6 independent culture series (0, 384, 744, 1224, 1824, and 2496 hours after starting the culture) are presented. Additional file 2: Figure S1. Expression changes of genes related to (a) arginine, (b) methionine, and (c) histidine biosynthesis pathways in tolerant strains with ethanol stress. Abbreviations: PRPP, phosphoribosyl pyrophosphate. Expression levels of 0 h in each gene represent the ones of parent strain. Asterisks (*) indicate expression levels of parent strain obtained absent ethanol stress as a reference. (PDF 4093 kb) Additional file 3: Figure S2. Correlations between gene expression changes for all possible pairs of tolerant strains. Each axis represents log_10_-transformed expression changes between a tolerant strain and the corresponding parent strain under ethanol stress conditions, while each dot represents the expression changes of a gene. Strain A had much amount of mutation, and strain C has large duplication, it might be the reason why strain A and C show lower correlation than other strains, (PDF 4093 kb) Additional file 4: Table S2. (CSV 7 kb)Intracellular metabolite concentrations in the parent and tolerant strains. Additional file 5: Figure S3. Metabolite concentrations in *de novo* and salvage purine biosynthesis. In each inset, the vertical axis shows the log-transformed absolute concentration (μM) under ethanol stress condition. Abbreviations: AMP, adenosine monophosphate; ADP, adenosine diphosphate; ATP, adenosine triphosphate; GMP, guanosine monophosphate; GDP, guanosine diphosphate; GTP, guanosine triphosphate; IMP, inosine monophosphate. (PDF 1031 kb) Additional file 6: Figure S4. Expression changes of genes related to the biosynthesis of phosphoribosyl pyrophosphate (PRPP) in tolerant strains under ethanol stress condition. Abbreviations: PRA, 5-phospho-β-D-ribosylamine; GAR, N^1^-(5-phospho-β-D-ribosyl)glycinamide; FAGR, N^2^-formyl-N^1^-(5-phospho-β-D-ribosyl)glycinamide; FAGM, 2-(formamido)-N^1^-(5-phospho-β-D-ribosyl)acetamidine; AIR, 5-amino-1-(5-phospho-D-ribosyl)imidazole; CAIR, 5-amino-1-(5-phospho-D-ribosyl)imidazole-4-carboxylate; SAICAR, (S)-2-[5-amino-1-(5-phospho-D-ribosyl)imidazole-4-carboxamido]succinate; AICAR, 5-amino-1-(5-phospho-D-ribosyl)imidazole-4-carboxamide; FAICAR, 5-formamido-1-(5-phospho-D-ribosyl)imidazole-4-carboxamide. (PDF 1110 kb) Additional file 7: Figure S5. Correlations between metabolite concentration changes for all possible pairs of tolerant strains. Each axis represents log_10_-transformed metabolite concentration changes between a tolerant strain and corresponding parent strain under ethanol stress conditions, while each dot represents the concentration changes of a metabolite. (PDF 1036 kb) Additional file 8: Figure S6. Sequence coverage of strain C. The number of mapped sequencing reads of Illumina analysis is plotted as a function of genome position. The coverage almost doubled in the region from 3,750,000 to 3,950,000 bp in the W3110 reference genome position, suggesting genomics duplication. The region includes 186 genes. No similar duplication was observed in other tolerant strains. (PDF 1032 kb) Additional file 9: Table S3. (CSV 11 kb)Identified mutations in the tolerant strain A. Additional file 10: Figure S7 Stability of ethanol tolerance. Strain F at the end point (2,500 h) and at 576 h was cultivated for 200 generations absent ethanol stress. After the cultivation, ethanol tolerance was evaluated by measuring specific growth rates in 5 % ethanol stress (red bars). The growth rates under ethanol stress were similar to those before the non-stress cultivation (blue bars) and were significantly higher than that of the parent strain. (PDF 976 kb) Additional file 11: Figure S8. Growth rates of isolated clones from the end-point populations of (a) strain E and (b) strain F. Growth rates of each 10 isolated clones and population under 5 % ethanol stress conditions are presented with that of the parent strain. Error bars indicate standard deviations calculated from three independent cultures. In both strain E and strain F, no significant difference was observed between the growth rates of population and clones (analyzed by one-way ANOVA). (PDF 969 kb) Additional file 12: Figure S9. Reproducibility of (a) transcriptome analysis, (b) metabolome analysis. Horizontal and vertical axis represents two repetitive measurements of the parent strain. In the transcriptome data, all data of repetitive experiments were within the range of 1.7 fold, while all metabolome data were within the range of 3 fold. (PDF 994 kb) Additional file 13: Table S4. (CSV 3 kb) Point mutations and indels found in the parent strains in comparison with W3110 reference genome. Additional file 14: Table S5. (CSV 3 kb)List of primers used in this study.
