# Supplementary figures and images for: Phenotypic convergence in bacterial adaptive evolution to ethanol stress
Source: BMC Evol Biol. 2015 Sep 3;15:180. doi: 10.1186/s12862-015-0454-6 (PMC4559166; doi:10.1186/s12862-015-0454-6)

(a)

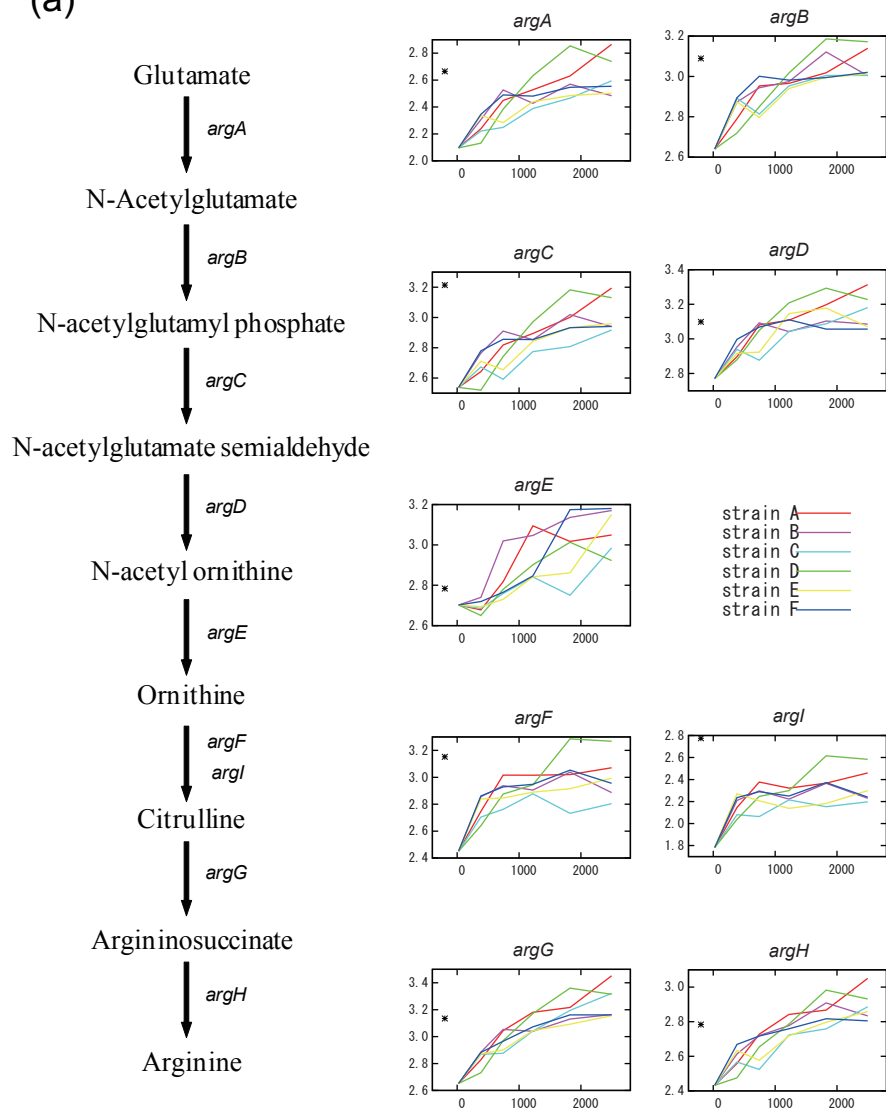

(b)

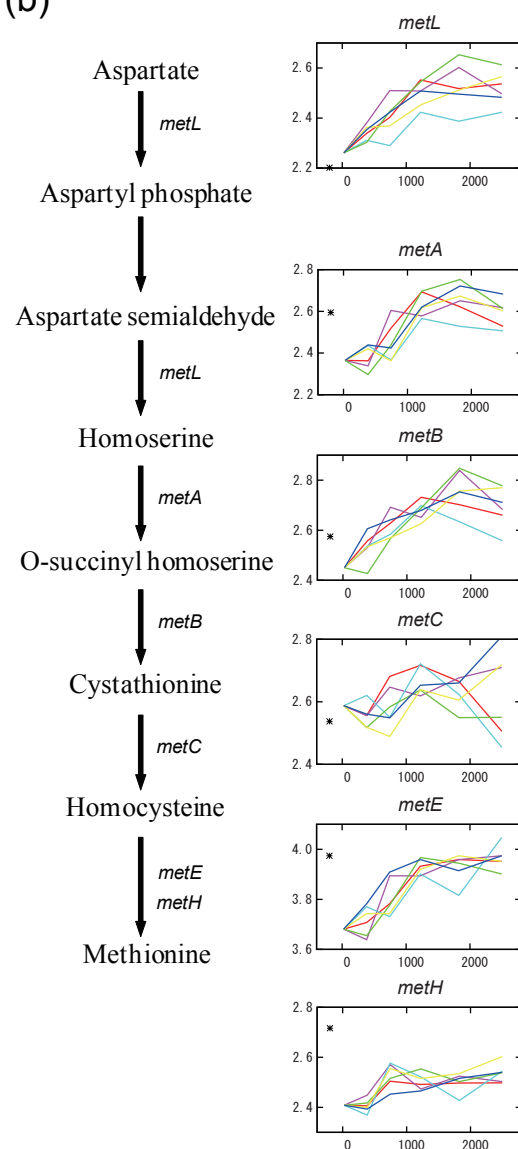

(c)

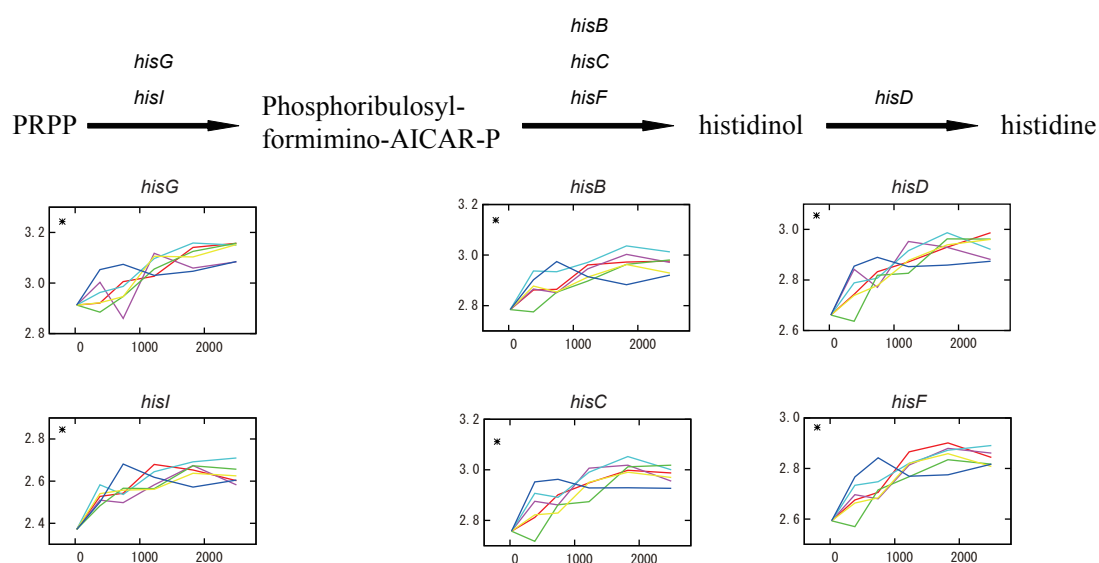

Supplement: Additional file 2: Figure S1. — Expression changes of genes related to (a) arginine, (b) methionine, and (c) histidine biosynthesis pathways in tolerant strains with ethanol stress. Abbreviations: PRPP, phosphoribosyl pyrophosphate. Expression levels of 0 h in each gene represent the ones of parent strain. Asterisks (*) indicate expression levels of parent strain obtained absent ethanol stress as a reference. (PDF 4093 kb) [file 12862_2015_454_MOESM2_ESM.pdf]

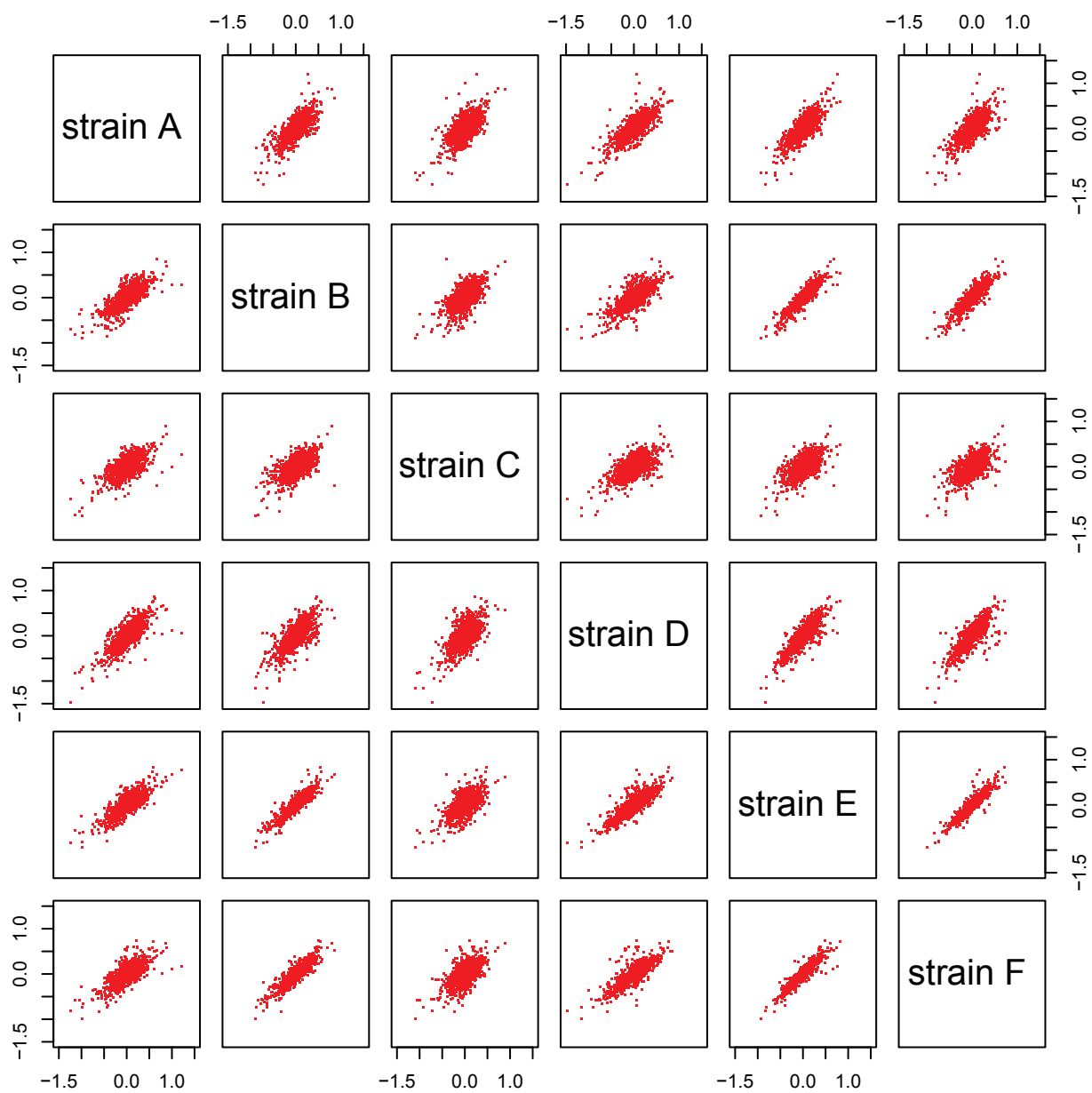

Horinouchi et al, Fig.S2

Supplement: Additional file 3: Figure S2. — Correlations between gene expression changes for all possible pairs of tolerant strains. Each axis represents log10-transformed expression changes between a tolerant strain and the corresponding parent strain under ethanol stress conditions, while each dot represents the expression changes of a gene. Strain A had much amount of mutation, and strain C has large duplication, it might be the reason why strain A and C show lower correlation than other strains, (PDF 4093 kb) [file 12862_2015_454_MOESM3_ESM.pdf]

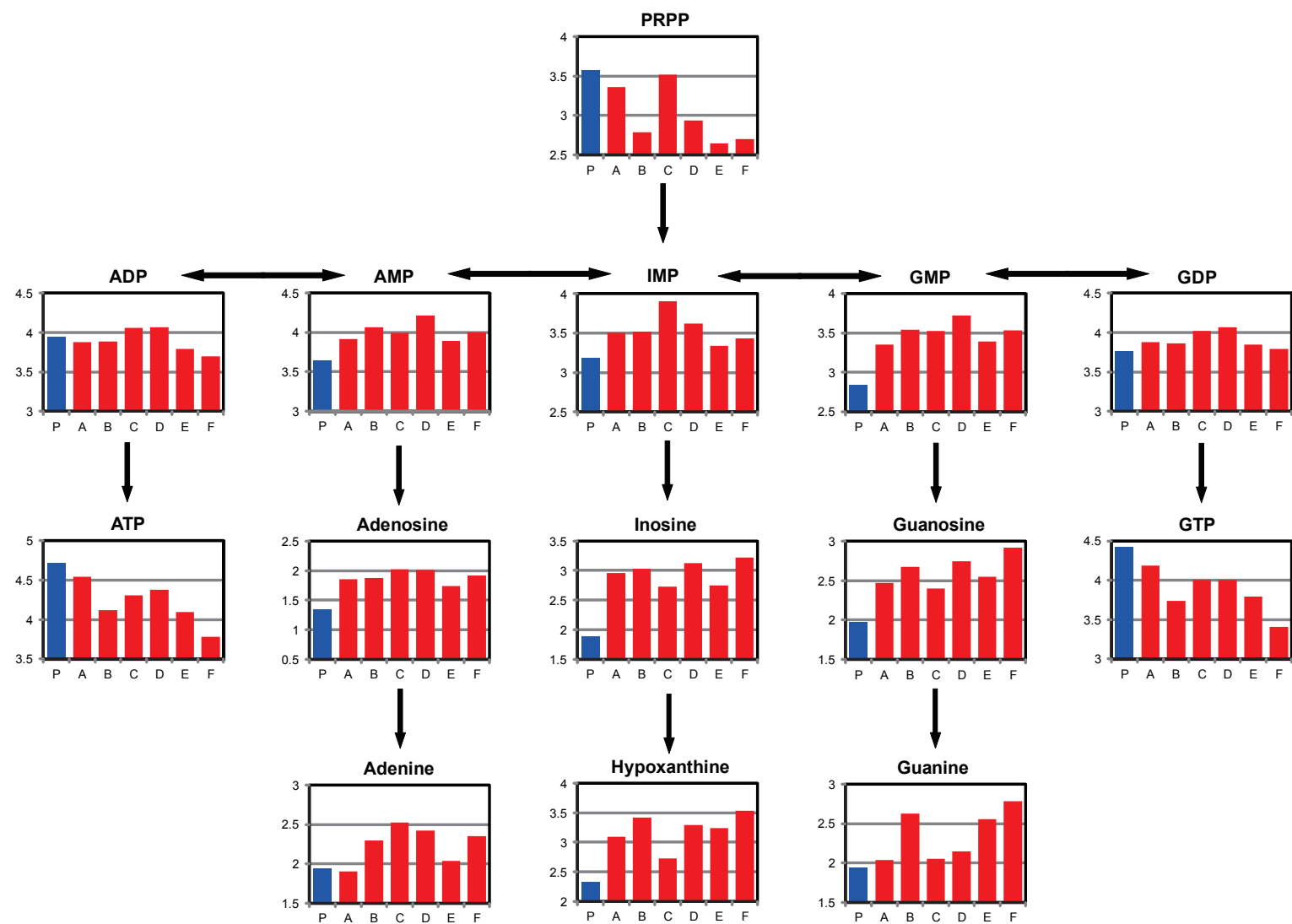

Horinouchi et al, Fig.S3

Supplement: Additional file 5: Figure S3. — Metabolite concentrations in de novo and salvage purine biosynthesis. In each inset, the vertical axis shows the log-transformed absolute concentration (μM) under ethanol stress condition. Abbreviations: AMP, adenosine monophosphate; ADP, adenosine diphosphate; ATP, adenosine triphosphate; GMP, guanosine monophosphate; GDP, guanosine diphosphate; GTP, guanosine triphosphate; IMP, inosine monophosphate. (PDF 1031 kb) [file 12862_2015_454_MOESM5_ESM.pdf]

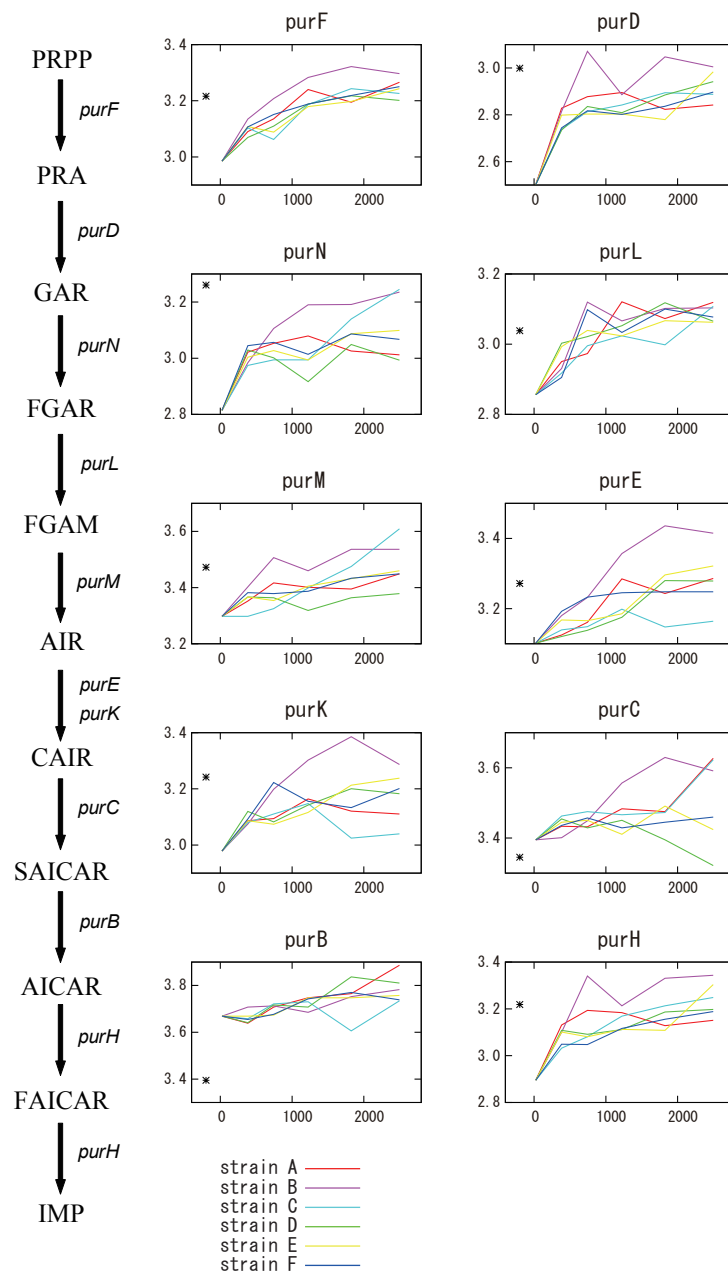

Horinouchi et al, Fig.S4

Supplement: Additional file 6: Figure S4. — Expression changes of genes related to the biosynthesis of phosphoribosyl pyrophosphate (PRPP) in tolerant strains under ethanol stress condition. Abbreviations: PRA, 5-phospho-β-D-ribosylamine; GAR, N1-(5-phospho-β-D-ribosyl)glycinamide; FAGR, N2-formyl-N1-(5-phospho-β-D-ribosyl)glycinamide; FAGM, 2-(formamido)-N1-(5-phospho-β-D-ribosyl)acetamidine; AIR, 5-amino-1-(5-phospho-D-ribosyl)imidazole; CAIR, 5-amino-1-(5-phospho-D-ribosyl)imidazole-4-carboxylate; SAICAR, (S)-2-[5-amino-1-(5-phospho-D-ribosyl)imidazole-4-carboxamido]succinate; AICAR, 5-amino-1-(5-phospho-D-ribosyl)imidazole-4-carboxamide; FAICAR, 5-formamido-1-(5-phospho-D-ribosyl)imidazole-4-carboxamide. (PDF 1110 kb) [file 12862_2015_454_MOESM6_ESM.pdf]

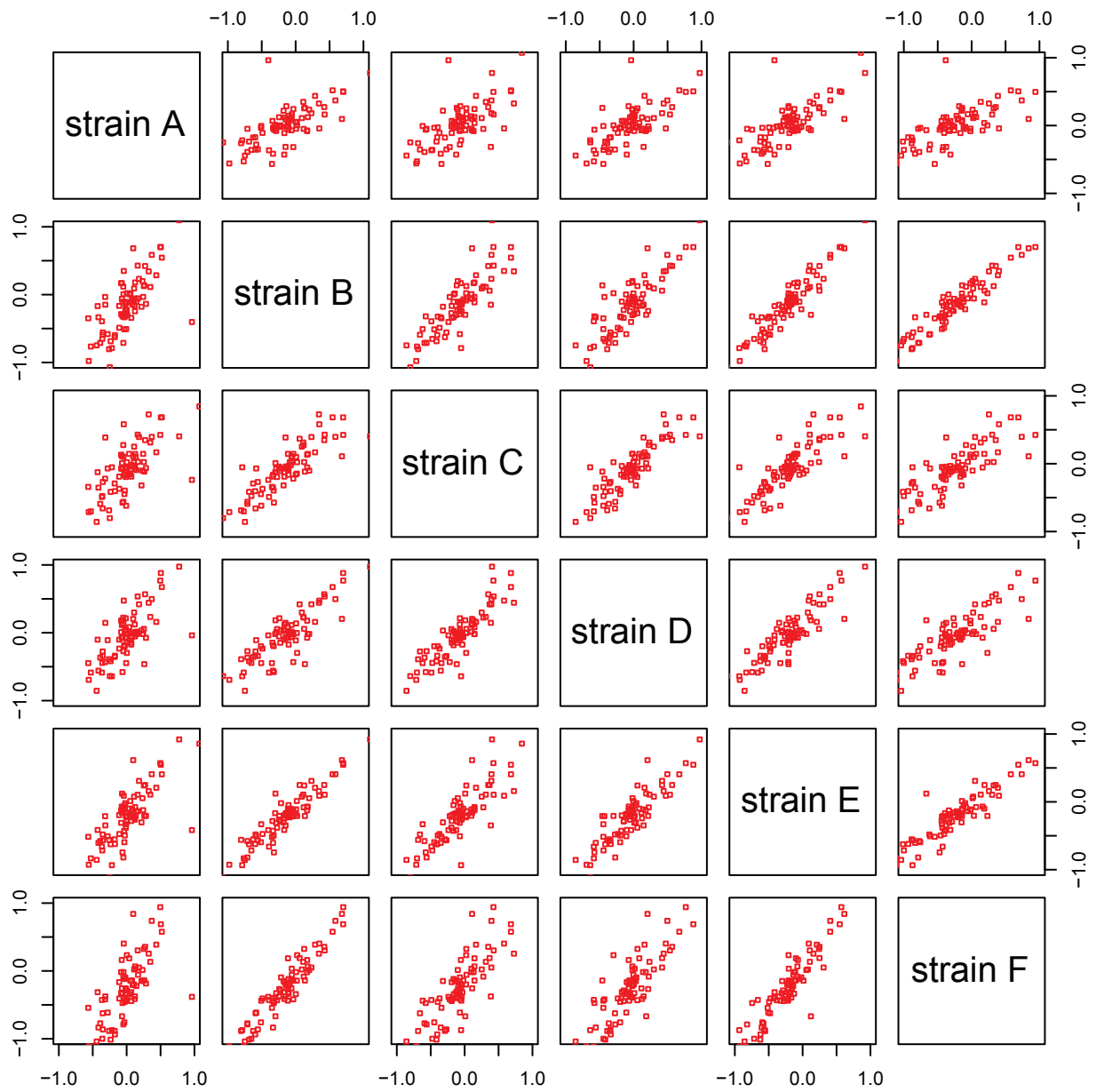

Horinouchi et al, Fig.S5

Supplement: Additional file 7: Figure S5. — Correlations between metabolite concentration changes for all possible pairs of tolerant strains. Each axis represents log10-transformed metabolite concentration changes between a tolerant strain and corresponding parent strain under ethanol stress conditions, while each dot represents the concentration changes of a metabolite. (PDF 1036 kb) [file 12862_2015_454_MOESM7_ESM.pdf]

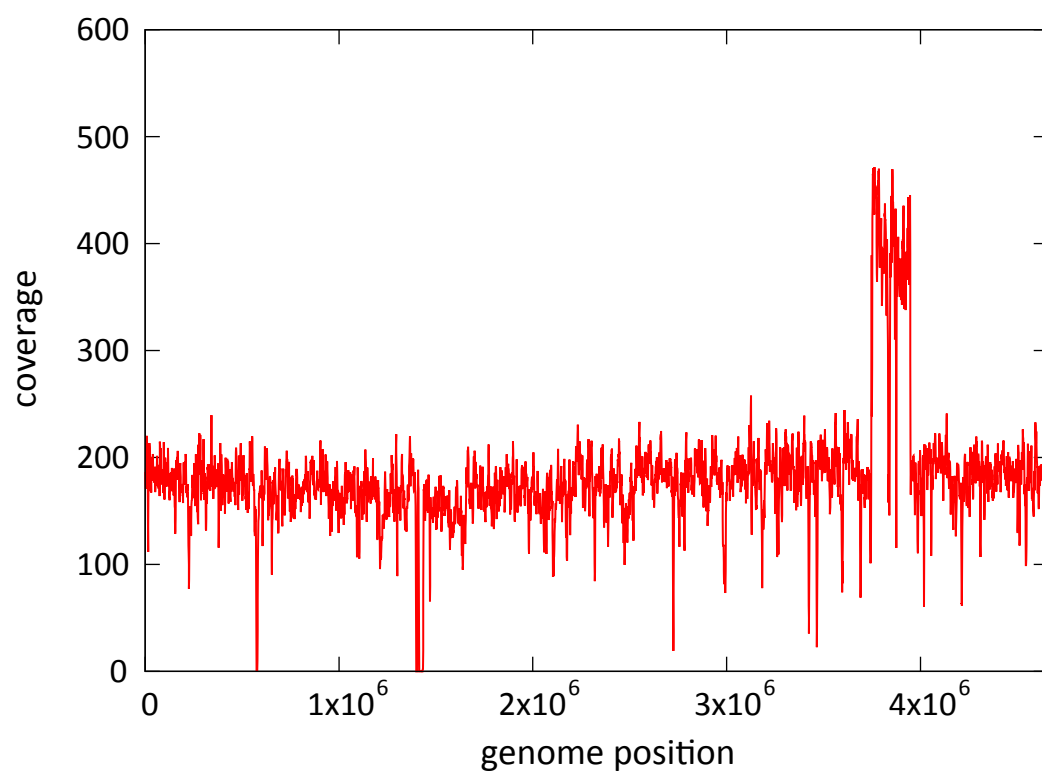

Horinouchi et al, Fig.S6

Supplement: Additional file 8: Figure S6. — Sequence coverage of strain C. The number of mapped sequencing reads of Illumina analysis is plotted as a function of genome position. The coverage almost doubled in the region from 3,750,000 to 3,950,000 bp in the W3110 reference genome position, suggesting genomics duplication. The region includes 186 genes. No similar duplication was observed in other tolerant strains. (PDF 1032 kb) [file 12862_2015_454_MOESM8_ESM.pdf]

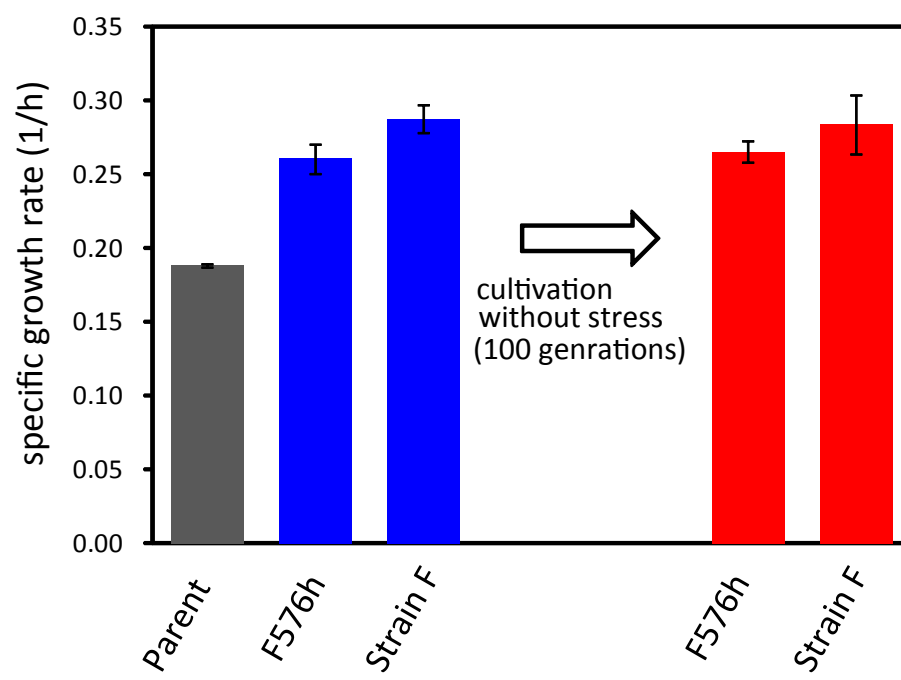

Horinouchi et al, Fig.S7

Supplement: Additional file 10: Figure S7 — Stability of ethanol tolerance. Strain F at the end point (2,500 h) and at 576 h was cultivated for 200 generations absent ethanol stress. After the cultivation, ethanol tolerance was evaluated by measuring specific growth rates in 5 % ethanol stress (red bars). The growth rates under ethanol stress were similar to those before the non-stress cultivation (blue bars) and were significantly higher than that of the parent strain. (PDF 976 kb) [file 12862_2015_454_MOESM10_ESM.pdf]

(a)

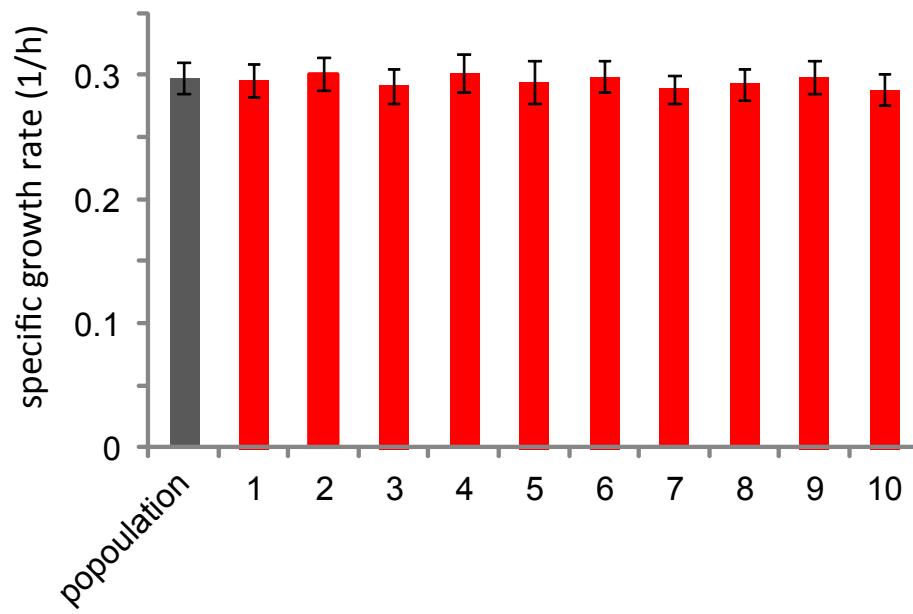

(b)

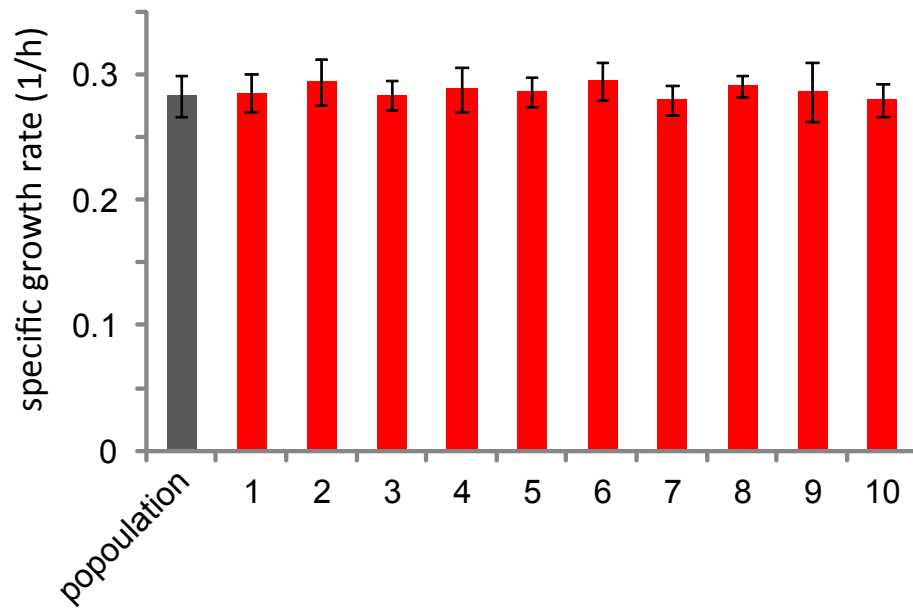

Supplement: Additional file 11: Figure S8. — Growth rates of isolated clones from the end-point populations of (a) strain E and (b) strain F. Growth rates of each 10 isolated clones and population under 5 % ethanol stress conditions are presented with that of the parent strain. Error bars indicate standard deviations calculated from three independent cultures. In both strain E and strain F, no significant difference was observed between the growth rates of population and clones (analyzed by one-way ANOVA). (PDF 969 kb) [file 12862_2015_454_MOESM11_ESM.pdf]

(a)

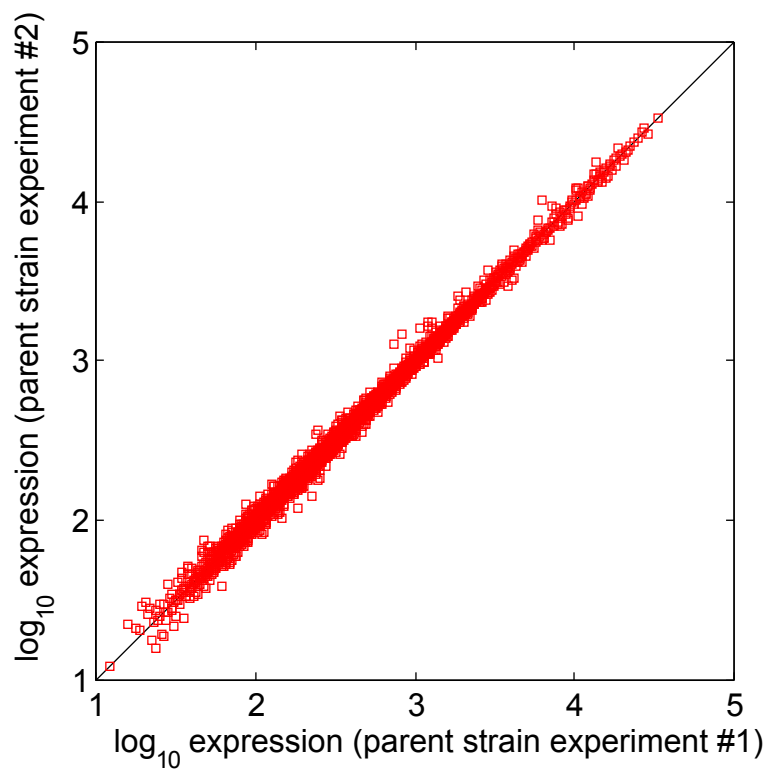

(b)

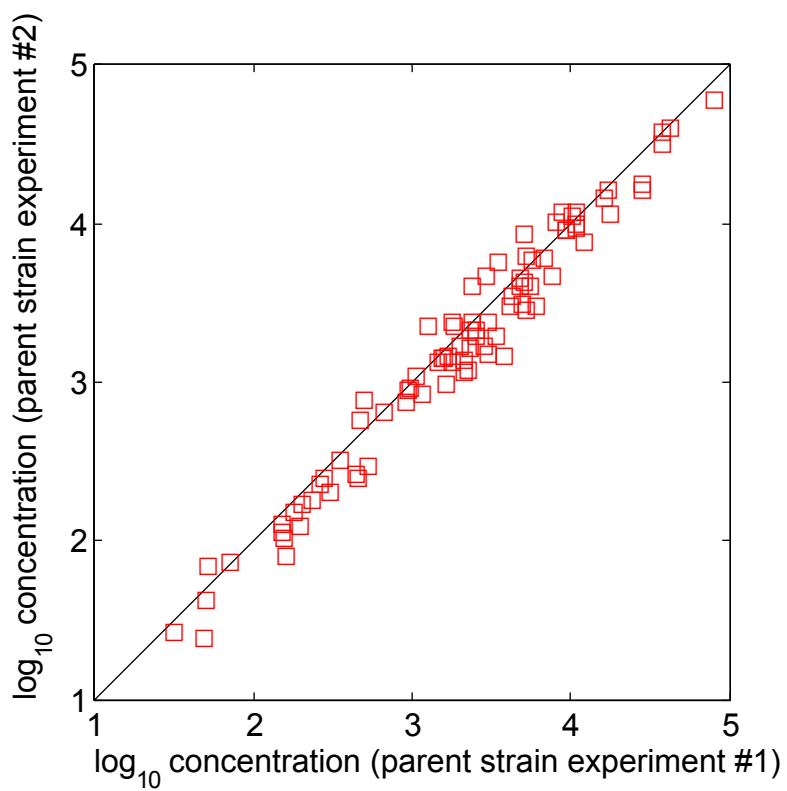

Supplement: Additional file 12: Figure S9. — Reproducibility of (a) transcriptome analysis, (b) metabolome analysis. Horizontal and vertical axis represents two repetitive measurements of the parent strain. In the transcriptome data, all data of repetitive experiments were within the range of 1.7 fold, while all metabolome data were within the range of 3 fold. (PDF 994 kb) [file 12862_2015_454_MOESM12_ESM.pdf]
